# A systematic review of the factors – enablers and barriers – affecting e-learning in health sciences education

**DOI:** 10.1186/s12909-020-02007-6

**Published:** 2020-03-30

**Authors:** Krishna Regmi, Linda Jones

**Affiliations:** 1 Faculty of Health and Social Sciences, Institute for Health Research, University of Bedfordshire, Luton, LU2 8LE UK; 2 Centre for Medical Education, School of Medicine, University of Dundee, The Mackenzie Building, Kirsty Semple Way, Dundee, DD2 4BF UK

**Keywords:** Health sciences, E-learning, Barriers, Enablers, Widening participation, Lifelong learning

## Abstract

**Background:**

Recently, much attention has been given to e-learning in higher education as it provides better access to learning resources online, utilising technology – regardless of learners’ geographical locations and timescale – to enhance learning. It has now become part of the mainstream in education in the health sciences, including medical, dental, public health, nursing, and other allied health professionals. Despite growing evidence claiming that e-learning is as effective as traditional means of learning, there is very limited evidence available about what works, and when and how e-learning enhances teaching and learning. This systematic review aimed to identify and synthesise the factors – enablers and barriers – affecting e-learning in health sciences education (el-HSE) that have been reported in the medical literature.

**Methods:**

A systemic review of articles published on e-learning in health sciences education (el-HSE) was performed in MEDLINE, EMBASE, Allied & Complementary Medicine, DH-DATA, PsycINFO, CINAHL, and Global Health, from 1980 through 2019, using ‘Textword’ and ‘Thesaurus’ search terms. All original articles fulfilling the following criteria were included: (1) e-learning was implemented in health sciences education, and (2) the investigation of the factors – enablers and barriers – about el-HSE related to learning performance or outcomes. Following the PRISMA guidelines, both relevant published and unpublished papers were searched. Data were extracted and quality appraised using QualSyst tools, and synthesised performing thematic analysis.

**Results:**

Out of 985 records identified, a total of 162 citations were screened, of which 57 were found to be of relevance to this study. The primary evidence base comprises 24 papers, with two broad categories identified, enablers and barriers, under eight separate themes: facilitate learning; learning in practice; systematic approach to learning; integration of e-learning into curricula; poor motivation and expectation; resource-intensive; not suitable for all disciplines or contents, and lack of IT skills.

**Conclusions:**

This study has identified the factors which impact on e-learning: interaction and collaboration between learners and facilitators; considering learners’ motivation and expectations; utilising user-friendly technology; and putting learners at the centre of pedagogy. There is significant scope for better understanding of the issues related to enablers and facilitators associated with e-learning, and developing appropriate policies and initiatives to establish when, how and where they fit best, creating a broader framework for making e-learning effective.

## Background

There are different meanings or interpretations of e-learning, but employing the technology to provide online access to learning resources for the improvement of learning is its principal aspect [1, 2]. E-learning has been defined as “an educational method that facilitates learning by the application of information technology and communication providing an opportunity for learners to have access to all the required education programmes” [3]. The term e-learning has been interchangeably used with the terms web-based learning, online learning or education, computer-assisted or -aided instruction, computer-based instruction, internet-based learning, multimedia learning, technology-enhanced learning and virtual learning [4, 5]. Such nomenclature has led to confusion as to whether e-learning is part of the medium (e.g. computer-assisted instruction) or the delivery mechanism (e.g. online learning).

There are different models or designs of e-learning which have been used in practice, the most common of which are: (a) enhanced or adjunct model – acts as an assistant in classroom face-to-face learning, providing relative independence to the students; (b) blended e-learning model – integration of classroom face-to-face learning experiences with online learning; and (c) pure online or fully-online model – without classroom or traditional face-to-face learning, to provide maximum independence to the students. This model can be further divided into individual and collaborative learning, with the collaborative learning option being sub-divided into synchronous (face-to-face) and asynchronous (text-based internet) [6, 7].

Similarly, there are different components of e-learning; for example, Ruiz et al. [5] describe three components: (a) development of content; (b) management of the content; and (c) delivery of the content in a synchronous or asynchronous way. Ruggeri et al. [8] highlight four components: (a) synchronicity (asynchronous vs synchronous); (b) location (same place vs distributed); (c) independence (individual vs collaborative); and (d) mode (electronic-only vs blended). Cook [9] also proposes four components: (a) mode of delivery of contents (e.g. textbook, face-to-face, computer-based, television); (b) configuration, i.e. differences within a given media format (e.g. web-based discussion board vs face to face, small-group discussion, lecture); (c) instructional method (e.g. learning activities, self-assessment questions, clinical cases); and (d) presentation (e.g. hyperlinks, multimedia, font simulation fidelity).

Recently, e-learning has been well recognised as mainstreaming in health sciences education (HSE) – medical, dental, public health, nursing, and other allied healthcare education – but the role of e-learning and its effect on learners’ performance or enhancing their learning has been well debated. E-learning, however, has had less impact than intended, and HSE practices have remained largely unchanged over the past decade. Cook et al. [4] raise some concerns over whether e-learning in medical education or el-HSE would actually enhance learning, particularly “the extent to which knowledge-based learning compared with alternative approaches to medical education”.

Though some published systemic reviews on e-learning have provided some promises that e-learning would be equally as effective as traditional methods of learning or teaching, still there is very limited evidence demonstrating when and how best e-learning enhances education and learning, and the factors associated with it [4, 10–14]. As Kim [15] argues, most of the published evidences, including the systematic reviews on e-learning, appear to have three major limitations: (a) they are mostly descriptive; (b) they have clearly failed to demonstrate the outcome measures; and (c) the majority have faults due to weakness or inappropriateness in study designs.

Another systematic review, capturing 176 empirical studies, conducted between 1996 and 2008, shows “students in online conditions performed modestly better, on average than those learning the same material through traditional face-to-face instruction” [16]. These interpretations, however, should be treated with caution, as the conditions and dimensions for both methods are not the same, particularly the learners’ and facilitators’ time spent on setting or accomplishing tasks, level of accessibility, and convenience [4, 17].

Two recent systematic reviews conducted by Du et al. [18] with nine RCTs, and Lahti et al. [19] with 11 RCTs, examined the effects of e-learning or web-based nursing education, but the findings reported were not significantly different between these two methods (e-learning and traditional education) as they reported almost similar results or slight improvements in knowledge, mainly on learners’ levels of satisfaction associated with e-learning. A Cochrane Review including 16 randomised trials (involving 5679 health professionals), published in 2018, examining the effects of e-learning versus traditional learning, reported little or no differences in patient outcomes or health professionals’ skills and behaviours [20].

Similarly, several studies make claims for e-learning and learning enhancement, but the results appeared rather mixed [4, 21, 22]. It has been found that if we simply compare the outcomes between e-learning and no training interventions, e-learning is generally far more effective in gaining knowledge, skills including positive behaviours, but this does not necessarily mean that the results are significant mainly due to the fact that results are heterogeneous (i.e. inconsistent results) and are frequently in small studies [23, 24].

Ellaway and Masters [25] also argue that “despite several decades of research and development in and around the use of computers in education, its practices and techniques are fluid and subject to change for more than other aspects of healthcare education”. However, strong evidence of el-HSE and its expectations and the factors – enablers and challenges – concerning e-learning is rather limited, scattered and patchy [15, 22, 26].

First, some evidence is available about the examining of e-learning from knowledge and attitudes of learners, but very limited evidence exists on the “impact of e-learning on learning outcomes” [27]. Second, limited studies have been published measuring e-learning and its effectiveness on education performance [22, 28]. Cook and McDonald [29] state that in e-learning, several questions have not yet been answered; for example, “what are the elements of effective e-learning in health sciences education (el-HSE), effective design and how do these vary for learners at different levels”, and there was no single paper published “on how to develop and implement e-learning interventions” effectively, as some forms of methodological innovation are required in education and learning to develop learners’ knowledge and skills both in academic and practice environments [13]. Third, recent research based on 72 studies also reported the lack of good research available on e-learning, and they pointed out that: “no studies have investigated e-learning in medical education (also in el-HSE) in a systematic way or with a focus on specific practice areas” [30].

In addition, several studies equally failed to capture the wider aspects of e-learning, exploring policy drivers, learning content, pedagogy methods, variability in designs and instructional methodologies, quality standards, or educational theories or principles [31, 32]. Though some studies have compared e-learning with traditional approaches of teaching/learning, results appeared conflicting and inconclusive [10, 33].

This systematic review, therefore, aimed to identify and synthesise the factors – enablers and barriers – affecting e-learning in health sciences education (el-HSE) that have been reported in the medical literature.

## Methods

This study utilised a systematic literature review (SLR) method. SLR is considered a valuable form of research, which also closely follows the principles of scientific methods, through being “designed to locate, appraise and synthesise the best available evidence” in relation to the research purpose, to be able to provide “informative and evidence-based” research [34].

### Search strategy

A systematic search for articles published on el-HSE was performed in MEDLINE, EMBASE, Allied & Complementary Medicine, DH-DATA, PsycINFO, CINAHL, and Global Health, from 1980 through 2019. Primary search terms were e-learning (all synonyms) and health sciences education (all synonyms) using ‘Textword searching’ – searching for a word or phrase appearing anywhere in the document, where the document is the citation (article title, journal name, author), not the full text of an article, and ‘Thesaurus (MeSH, EMTREE) searching’, employing Boolean operators and truncations, such as (“e-learning” OR “online learn*” OR “distance learn*” OR “computer-assisted instruction” OR “web-based learning” OR “internet-based learning” OR “multi-media learning” OR “technology-enhanced learning” OR “distributed learning” OR “virtual patients” OR “virtual microscopy” OR “virtual environment” OR “virtual learning”) AND (“continuing medical education” OR “medical education” OR “health sciences” OR “basic Sciences” OR “public health education” OR “nursing education” OR “public health nursing” OR “allied health education”) AND (“challenges” OR “barriers” OR “enablers” OR “facilitator*”). A detailed systematic review protocol, developed by the authors, with specific search terms has been provided in the Additional file 1.

### Inclusion and exclusion criteria

All original research articles on e-learning fulfilling the following eligibility criteria were included:

- e-learning was implemented in health sciences education
- the investigation of the enablers and barriers about el-HSE related to learning performance or outcomes
- articles were published in peer-reviewed journals, in English, after 1980.

Exclusion criteria were:

- articles falling outside e-learning in health sciences education
- articles published in secondary, non-empirical studies or grey literature
- commentaries, review documents, case studies, letters, discussion papers, posters, conference abstracts, congress reports and dissertations
- articles not published in peer-reviewed journals
- no full text available.

### Selection of studies

**Fig. 1:**
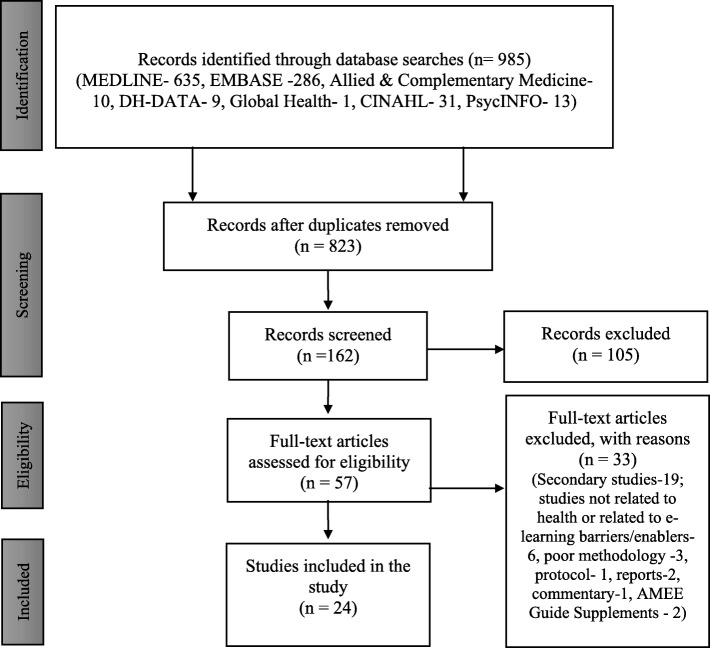
PRISMA Flow diagram to show results of searches

The citations identified through the searches was imported into Refworks software (https://www.refworks.com/). The literature which emerged from the databases, snowballing and hand-searching has been screened at two stages: first, a review of abstracts and titles of the retrieved literature to see whether they meet minimum inclusion criteria. Second, the full text of the included articles was reviewed and retrieved using a critical appraisal tool [35]. As Means et al. [16] argue, “the intent of the two-stage approach was to gain efficiency without risking exclusion of potentially relevant, high-quality studies of online learning effects”. The standard PRISMA flowchart has been used to provide the process of study selection [36] (Fig. 1).

### Data analysis and synthesis

Based on the final search outputs, 24 papers were identified to fit in the review, and they were mostly quantitative in nature. As Clarke [37] argues, when such heterogeneity in methodology exists, “systematic review does not need to combine the results of the studies to provide an average estimate”. Therefore, in this study, data were synthesised through narrative synthesis using thematic analysis (TA) [38]. TA has been considered as “a method [ …] identifying, analysing and reporting patterns (themes)” or meaning searching across the literature or data [39]. In this study, to identify the recurrent themes we followed these six steps when synthesising data using thematic analysis, e.g. familiarising with the data, developing initial (sub) codes, searching for (sub) themes, reviewing (sub) themes, charting or compiling ideas or issues, and producing final data in line with the study aims and objectives [40, 41].

### Tabulating the included studies

We developed a table to capture the nature of the studies (designs, methods and populations, see Table 2). To ensure the accuracy and completeness of the data extraction by KR, data extraction was checked by LJ. As Petticrew and Roberts [65] confirm, this would not only improve the process of transparency by better understanding what sorts of “data have been extracted from which studies”, but also recognising the “contribution made by each study to the overall synthesis”.

### Quality assessment

Methodological quality of the included studies was assessed with the ‘*QualSyst* developed by Kmet and colleagues [35], and we particularly found its scoring system useful because it has clearly shown the process to be more “systematic, reproducible and quantitative means of assessing the quality” of those retrieved papers [66]. There are checklists of 14 question items for assessing quantitative and 10 questions for qualitative studies, and a score of 0–2 has been awarded to each item, with a final score calculated by summating the total score across the items and dividing them by the total possible sum (e.g. 28 for quantitative and 20 for qualitative studies) [66, 67]. A cut-off of 75% as the threshold for quantitative, and 55% for qualitative papers has been set up. For mixed methods studies, specifically designed questions were employed to assess the quality [67]. Complete details regarding quality appraisals of individual studies were provided in the Additional file 2.

## Results

Out of 985 records identified, a total of 162 citations were screened, of which 57 were found to be of relevance to this study. The primary evidence base comprises 24 papers; most were quantitative (14, 58.33%) in design (see Fig. 1). Most studies were published between 2005 and 2019. This is based on approximately 2355 participants, who were mostly undergraduate and postgraduate students or learners (1831, 77.74%). The geographical range of papers covered mostly the high-income regions. Detail information regarding the demographic profile of included articles are presented in Table 1. A summary of excluded studies and reasons for exclusion are provided in the Additional file 3.

**Table 1:** Demographic profile of included studies

Study characteristics	Number of studies (%)
***Year of publication***	
• 2005–2008	7 (29.16%)
• 2009–2012	5 (20.83%)
• 2013–2015	6 (25%)
• 2016–2019	6 (25%)
***Type of study***	
• Quantitative	14 (58.33%)
• Qualitative	7 (29.16%)
• Mixed methods	3 (12.5%)
***Study sample***	
• 50 and <	8 (33.33%)
• 50–100	7 (29.16%)
• 100–150	5 (20.83%)
• 150–200	–
• 200–250	1 (4.16%)
• 250–300	1 (4.16%)
• 300–350	1 (4.16%)
• 350 and >	1 (4.16%)
***Countries***	
• Australia	2 (8.33%)
• Belgium	1 (4.16%)
• Brazil	1 (4.16%)
• Canada	1 (4.16%)
• Germany	1 (4.16%)
• Iran	2 (8.33%)
• Netherlands	2 (8.33%)
• Norway	1 (4.16%)
• Slovenia	1 (4.16%)
• Spain	1 (4.16%)
• Sweden	1 (4.16%)
• UK	6 (25%)
• USA	3 (12.5%)
• Pakistan	1 (4.16%)

Upon conducting thematic analysis of the included studies, it was possible to obtain two broad descriptive themes/categories: enablers or drivers of, and barriers or challenges to, el-HSE, under which eight important themes have emerged.

### Enablers or drivers

- Theme 1. Facilitate learning
- Theme 2. Learning in practice
- Theme 3. Systematic approach to learning
- Theme 4. Integration of e-learning into curricula

### Barriers or challenges

- Theme 5. Poor motivation and expectation
- Theme 6. Resource-intensive
- Theme 7. Not suitable for all disciplines/contents
- Theme 8. Lack of IT skills

### Enablers or drivers

#### Theme 1: Facilitate learning

Seventeen out of 24 studies reported that e-learning has been one of the successful approaches and tools to facilitate the process of learning amongst healthcare professionals in practice [3, 42, 43, 45, 46, 48, 50–53, 55–59, 61, 62]. Several studies highlighted that e-learning has been influenced mostly by structured frameworks in the way it is contextualised, builds in the learners’ experience, and aligns with course assessments or learning outcomes [45, 50, 56].

In addition, it develops appropriate interaction between and amongst learners and facilitators that would enhance learning by making it more integrated and contextualised, with the possibility of bringing learners a high level of exciting and stimulating learning [46, 50]. The following two extracts illustrate this:

E-learning provides the opportunity for instructors or teachers to teach better, since it allows them to use a range of both technical and pedagogical teaching tools [62].

Integrated, clinically-oriented interdisciplinary learning that focuses on knowledge and skills to encourage “learning through doing” is an important attribute of online e-learning [46].

Studies also noted that when learners wish to learn in a more in-depth way about the practice or the context, then e-learning would be a preferred approach to learning as it considers four components – content, educator, system, and learners – which might play an important part in making e-learning effective [45, 50, 53]. It has now become an accepted tool or approach for continuing professional development (CPD), mainly among medical, nursing and allied healthcare professionals, as the nature of e-learning often benefits from contemporary information, delivered rapidly and flexibly, adopting varying formats [61, 62].

Flexibility in nature means that learning often takes place at the learners’ own pace, regardless of their geographical locations, and materials can be accessed any time, and these are reported as perceived benefits or key enablers compared with lecture mode [42, 46, 51, 52]. The extracts below highlight very clearly the strong support to the flexibility aspect of e-learning:

I think online also you kind of get a more in-depth amount of information because you can read it yourself at your own time rather than having to fit a certain amount of information into like a one-hour, two-hour lecture. You can spend like half an hour blocks trying to get that information. So it’s all [set] out there for you and it’s really well explained, whereas someone in a lecture has only a certain amount of time to kind of go over it … and with less detail than what you can get online [46].

#### Theme 2: Learning in practice

Fifteen out of 24 studies included in this review reported that e-learning had been found an effective approach regarding the transformation of knowledge-integration into practice through education and training, including CPD in healthcare settings [42–44, 46–57, 59]. A study conducted among 148 GPs, using exploratory factor analysis, has highlighted that the intention of using el-HSE was mainly due to its widely accepted and preferred method in practice [49]. Similarly, Morente et al.’s [57] study conducted among nursing students has also reported that e-learning has improved educational efficacy and better learning acquisition.

Several studies further showed that e-learning is the most effective approach for transferring clinical skills and knowledge, using virtual clinical case studies adopting a mixed learning approach, combining different styles and modes not only to facilitate learning but also to bring positive change in practice [47, 53–55]. Therefore, the integration of theoretical learning into practice using el-HSE is evident [44, 47, 53, 56].

Six out of 24 papers highlighted that developing learners’ motivation, satisfaction, expectation, training and support needs were the key reported factors for improving working practice [42, 43, 46, 48, 54, 58]. Similarly, they also noted that effective information and education support, ease of access, inter-professional learning, learning appropriately, integrating and applying learners’ values and skills acquisition into practice were consistently highlighted in the retrieved papers as successful for bringing positive impacts on work and opportunities to learn. Gormley et al.’s [48] survey conducted amongst undergraduate medical students, assessing the effectiveness of e-learning in clinical skills, also demonstrated that learners found e-learning particularly useful as learners would be able to access and review e-learning materials before their learning. Second, it also “encouraged them to see real patients through uploading relevant online videos of particular use which appeared as one of the important domains in their learning” [48]. The relevant extract below highlights the support of e-learning:

This method [e-learning] of teaching as being a good way to address sensitive consultations, important issues and bringing attention to situations [even] they [students] may not have encountered [51].

#### Theme 3: Systematic approach to learning

Eleven out of 24 papers showed that el-HSE is a superior approach to classical or traditional learning in terms of improving quality of education through integrating theoretical contexts into practice [3, 42–44, 48, 49, 51, 55, 56, 58, 59]. Accompanying these thoughts, a recent study conducted among undergraduate healthcare students using a mixed-methods approach reported that the nature of e-learning often adopts some systematic approach to learning, i.e. moving from simple to complex learning, arguing that ideas or knowledge are logical and interconnected for the consolidation of learning, from the holistic perspective, to meet the learning goals [58]. Such a process would also help to create a social construction of knowledge [59].

As e-learning involves multidisciplinary uses, creativity, motivation, quality and accessibility, it provides an alternative education approach or opportunity for lifelong learning, addressing both the long-term and short-term healthcare and education goals of learners [3, 43, 44, 50, 55, 56]. It has also been reported that the effectiveness of e-learning would be determined by the extent to which learners’ short-term and long-term personal and professional educational needs are met [3, 49, 55].

Similarly, several authors reported that in a new era, the traditional style of teaching and learning is getting out-of-date, so e-learning would be an appropriate means and end for lifelong learning, mainly due to its nature of flexibility in education and learning [48, 56, 58, 59].

#### Theme 4: Integration of e-learning into curricula

Six out of 24 studies reported some pedagogic issue associated with learning methods and styles [44, 47, 50, 55, 56, 64]. Curriculum and pedagogy are interlinked by two different approaches (models), e.g. blended learning and flipped classroom as they present some degree of the interface between learners and philosophy of the learning. Blended learning intervention is simply integration of classroom face-to-face learning experiences with online learning to facilitate independent, interactive and collaborative learning due to its flexible and technologically rich format. This approach is, however, reported as complex and challenging in nature due to its different possible designs, and contextual needs. Flipped or inverted classroom is a form of blended learning, where students learn in part in class, and in part through online learning, providing students more choices in terms of the place and pace of learning experiences. The key factors for success in these models of e-learning or online education are to collaborate and integrate e-learning into current curricula [51, 55].

Several studies also reported that engagement in e-learning among learners and professionals, mainly from HSE, is useful and has positively impacted engagement and retention of learning [50, 58, 63]. The extracts below illustrate this:

The success for medical educators is to ensure engagement with the online self-directed component of the module. This initial learning should allow students to fill in the gaps in their knowledge by focusing them on what they do not know, which may enhance retention [58].

Accompanying these issues, four papers have consistently reported that contacts (with learners and facilitators), opportunities for self-assessment, flexibility, and faster and easier access to quality learning resources are equally important dimensions or approaches that would enhance students’ interest in learning and improve both their level of engagement and their learning autonomy [3, 44, 50, 56, 64].

### Barriers or challenges

#### Theme 5: Poor motivation and expectation

Seven out of 24 papers reported factors that may be variants to learners’ motivation and expectations to be able to meet their personal and professional needs and goals [44, 45, 47, 50, 55, 56, 60]. While analysing reported factors, two groups of factors appeared common, i.e. internal and external factors. Internal factors refer to the poor engagement, poor perception and motivation, limited flexibility, high levels of anxiety and stress, lack of students’ self-discipline and low self-efficacy, as well as poor interactions between learners and facilitators. Such factors not only hinder the process of learning and motivation but also fail to meet learners’ healthcare needs and expectations [44, 45, 47, 56].

External factors are mostly related to the course structure, poor pedagogical design, clarity of the purpose and goal, education management policy, educational paradigms, learners’ diversity, current and future education workforce needs, financial independence, influence of national and international policies, lack of learning space, limited use of technology in education, poor evidence-based education and training, and strategic change in higher education as well as inadequate support [3, 45, 48, 50, 52, 53, 562–58, 65].

#### Theme 6: Resource-intensive

Nine out of 24 papers included in this review have reported that e-learning is a time-, cost- and labour-intensive approach [44, 45, 47, 50, 53, 55, 56, 60, 63]. Several papers also raised the technological or IT challenges, as several learners are not familiar with e-learning and in some contexts, even basic IT knowledge and skills are lacking [44, 45, 53, 55, 56, 63]. Thus, inappropriate equipment and technological illiteracy have raised some concerns with regard to the usefulness of el-HSE [59].

Both Hammarlund et al. [50] and Ikram et al. [53] also highlighted that issues related to long-term costs and resources raised concerns related to quality, usability and effectiveness, poor consideration of users’ needs, lack of time, and lack of students’ self-discipline, all of which would have a negative impact on e-learning.

#### Theme 7: Not suitable for all disciplines or contents

Eight out of 24 papers reported that integration of learning into the existing curricula would be problematic, as some disciplines, for example, biomedical, would take extensive time for learners and facilitators to adapt the content into e-learning curricula [44, 46, 50, 51, 55, 56, 59].

Additionally, several papers have reported that in e-learning, not only might some content not be suitable as these disciplines need practical or demonstrative types of learning, but also this creates some problems of communication, as well as a lack of group dynamics [44, 47, 50, 55]. The extract below illustrates this:

A few students felt that communication skills and reflective learning could not be taught by any method, and that motivating people who are not interested in communicating is the problem [51].

Gardner et al. [46] and Gensichen et al. [47] also raised some concerns related to quality – depth and breadth of learning, motivation as well as usability and effectiveness, and this can be seen from the following extract.

The risk of combing for answers to quizzes instead of active, comprehensive learning of information ‘… if I had a lot of these to do [e-learning learning packages] then I would very likely procrastinate or skim read through them or not pay as much attention because it’s not – I’m not necessarily having someone there … I might do it later or do it at this time,’ and it just keeps getting put off’ [46].

#### Theme 8: Lack of IT skills

Eight out of 24 papers identified the lack of IT or user-friendly IT as one of the key challenges of making e-learning successful in HSE [42, 44, 52, 53, 55, 56, 59, 61]. The extract below illustrates this point:

Lack of computer skills has been identified as a major barrier preventing doctors from using computer-based learning methods, rather than a lack of preference for new technologies [52].

**Fig. 2:**
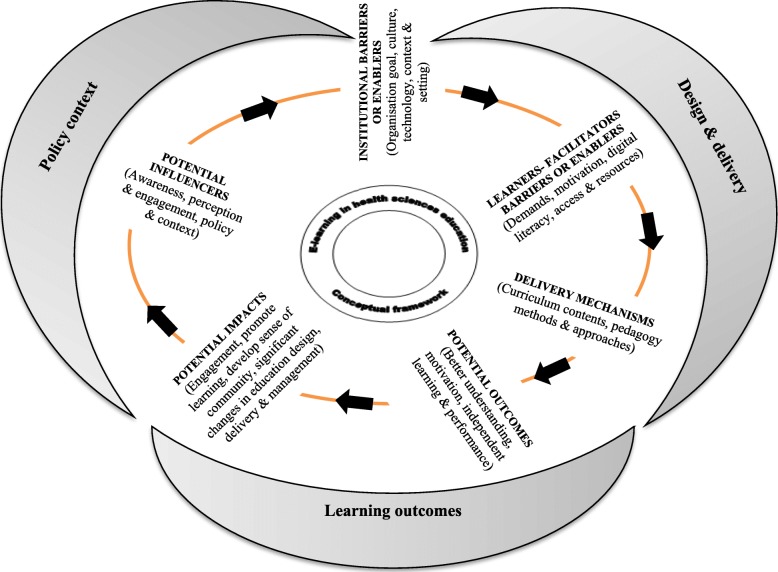
Conceptual framework of factors influence e-learning in health sciences education

Figure 2 is a conceptual framework (CF) that emerged while analysing the findings from those 24 papers. The CF characterised as a step-wise process in a circular flow involving six major components: a) potential influencers, b) institutional barriers or enablers, c) learners or instructors – barriers or enablers, d) delivery mechanisms, e) potential outcomes and f) impact under three broad categories i.e. institutional policy context, instructional design and delivery, and learning outcomes (improve achievements and learning engagement; develop a sense of community). This framework can also be called ‘e-learning CDDO (context, design, delivery and outcomes) configuration framework’ in HSE. This framework has sufficiently mapped the connections between e-learning and its outcomes, reflecting the learning context, potential influencing factors, reported enablers, barriers, and delivery mechanisms associated with instructional design and delivery, and the overall learning outcomes in relation to making el-HSE effective, from the perspective of learners, facilitators and professionals.

The most frequently recorded enablers included individualised and contextual learning, integration of theoretical learning into practice, and interactive, collaborative and flexible learning. The most frequently reported barriers also included course structure, learning space, isolation, poor institutional design, as well as time-, cost- and labour-intensive work obligations. Influence of national and international policies, organisational objectives and goals, learning management system, quality, standards and awareness, leadership and financial independence are some of the reported wider influencers. Similarly, increased knowledge, performance, learners’ freedom, engagement, and learning contribution to meet the needs of learners’ current and future healthcare needs to be described or recorded under the component of outcomes or wider impact. Table 2 provides a summary of factors which emerged from the findings identified about the components of the e-learning conceptual framework in health.

**Table 2:** Study characteristics and summary of the factors associated with e-learning

Study (First author and year), country	Focus of study	Stated research methods and anlysis	Participants	Categories reflect factors (i.e. policy & context, instructional design and delivery, and learning outcomes) influencing (the implementation or utilisation of) e-learning in HSE framework components				
				Potential influencing factors	Reported drivers or enablers	Reported barriers or challenges	Delivery mechanisms	Wider impact
Golband 2014 [3] Iran	Factors influencing e-learning	Analytical cross-sectional; ANOVA and Scheffe tests.	Postgraduate students (*n* = 60)	Education approach; e-learning global era in education	Consider four components of effective learning – learning content, educator, system and learner. Address learners’ backgrounds, motivations and expectations and needs	Not reported	E-learning of medicaleducation	Improved learning – learning content, educator, system and learner, improved motivations and met expectations, addressed learners’ short-term and long-term education needs in HSE.
Beckman 2008 [42] Belgium	E-learning and classification of pressure ulcers	Repeated measure design – experimental; chi-square tests	Nursing students (*n* = 236, 212 experimental and 214- control)	Evidence-based learning and education	Flexibility, quality, flexibility and costs, learning takes place regardless of learners’ geographical location and time	Labour-intensive, poor computer skills, demands high level of educational skills/experience	E-learning	Improved knowledge and performance, improved learning and application in practice
Boye 2012 [43] Norway	E-learning and medical immunology	Quantitative study; Mann-Whitney U-test	UG medical students (*n* = 73; 30 expt. And 43- control)	Popular approach in medical education, advancement of information technology in HE	Flexibility, improve learners’ reasoning and understanding skills.	Poor/lack of participation	E-learning	Students well appreciated e-learning, improved examination performance and outcomes, improved users’ satisfaction
Docherty 2006 [44] UK	E-learning - enablers or facilitators	Qualitative methods (Evaluation methods using a mixed methods)	Students *n* = 30 (face-to-face), 5 GPs, 2 nurses	Healthcare education and service provision, learning embeds multifaceted values, national plan – NHS, lifelong learning	Practice influenced learning, learning place and time, minimal involvement, flexible, integration with commitments, value and skills acquisition	Poor support, limited resources, lack of IT skills, isolation, lack of students’ self-discipline, isolated learning, resource-intensive costs and learning resources	E-learning in CDP	Increased knowledge and accessibility; increased flexibility. Improved working practice, improved learners motivation, expectation; improved knowledge and skills, flexible learning and integration
Gagnon 2007 [45] Canada	E-learning - barriers	Qualitative methods; theoretical framework	Physicians (*n* = 40)	Principles of evidence-based medicine, influence of the current paradigm of best clinical practice	Motivation, individualised flexible learning, tutors/peers support and feedback, flexible learning	Time constraints, poor technology, poor planning and delivery, lack of sufficient feedback, lack of familiarity with IT/computers, poor integration of learning in practice	E-learning in evidence-based Medicine	Increased learners’ motivation and self-discipline, received individualised support/feedback, improved learning and flexibility in practice.
Gardner 2016 [46] Australia	E-learning and students’ perspectives	Qualitative study, grounded theory	Physiotherapy students (n = 23)	Capacity – health workforce development	Flexibility – cost and time, motivation for learning, easy access to materials, encourage learners to learn through doing	Interactive learning, e.g. embedding quizzes or discussions, case-studies, real examples, would make learning effective	E-learning	Enhanced students’ learning; improved students’ attitude towards working in an inter-professional team; suitable for learning for both interdisciplinary and multi-disciplinary health professionals
Gensichen 2009 [47] Germany	E-learning and primary care	Modified Delphi - Quantitative study; descriptive stats	Primary healthcare experts (n = 60)	Education and learning are mainly financially independent in the healthcare industry	Workplace learning practice, clinical knowledge transfer in practice; embedding different styles or modes of learning (mixed learning) to suit learners’ learning needs/demands.	Resource-intensive – time and cost, problems of integration of learning into the existing academic and professional curriculum; fear of replacing traditional methods of teaching and learning; often failing to consider learners’ needs and interests	E-learning in primary care education	Improved learning; increased clinical knowledge using virtual clinical case studies in practice
Gormley 2009 [48] UK	E-learning and clinical skills	Survey; t-test and multiple regression analysis	UG medical students (*n* = 304)	Established method for teaching in medical schools, technological advancement in academic education	Facilitates learning, helpful for revision and exam preparation, motivates to learn, saving costs as learners don’t need to purchase textbooks	Potential isolation of learning, learners need to engage more to learn better, learners need to be familiarized with different modalities of teaching	E-learning	Reported confident, encouraged learners to attend the clinical attachment, standardized teaching, found useful in revising work, better performance in clinical skills
Hadadgar 2016 [49] Iran	E-learning and GPs - medical education	quantitative study; Kurtosis and skewness tests	GPs (*n* = 148)	Technology advancement, provisions of CPD in academic and healthcare industry	Satisfactory accessibility, increase flexibility	Problem of access to computers, time constraints, and unfamiliarity with computers	E-learning	Acceptance of e-learning in CPD
Hammarlund 2015 [50] Sweden	Factors influencing e-learning	Qualitative; content analysis	Undergraduate physiotherapy students (*n* = 34)	Both factors - external and internal influence learning including pedagogical design, collaborative learning in HSE.	Contextualised learning, learning aligned with course learning outcomes, appropriate assessments; opportunity to interact with teachers and peers, feedback from tutors, flexibility	Poor instructional design, poor course structure, limited learning space, poor relationship with peers and tutors, limited engagement, motivation, inflexibility, anxiety and stress, and low self-efficacy	Self-directed learning online	Increased healthcare learners’ knowledge, performance and progress; learners ‘freedom’ and motivation; improved current and future professional development in healthcare practice
Hawthrone 2009 [51] UK	E-learning and delivery modes	Evaluation methods, quantitative; Kruskal-Wallis and chi-square tests	Final year UG medical students (*n* = 223)	Not reported	Self-directed learning; learning tailored to individual students’ needs and preferences; convenience of learning, i.e. choose learners’ own time and place	Required appropriate knowledge in technologies to make effective learning, need to understand how people learn, poor teaching design/choice of tools	E-learning	Improved students’ learning, improved students’ performance, learning appeared relevant in practice, self-directed learning, and provoked thinking.
Hugenholtz 2008 [52] Netherlands	E-learning and continue professional development for occupational physicians	Randomised control trials – 4 blocks randomisation, t-test and chi-square tests	Physiotherapy students (n = 23)	E-learning is an effective teaching approach in continuing medical education, integration of internet technologies into CME programmes	Learners can choose any time and any geographical place for learning; useful for professional education and development in practice	None discussed	E-learning	Enhanced or sustained knowledge gains and improved learners’ behaviour change as compared to traditional learning/teaching approaches
Ikram 2015 [53] Netherlands	Developing effective module	Quantitative method; paired t-test	4th year medical students (*n* = 281)	Quality of care, equitable healthcare services, teaching patient diversity in medical curricula	Learning in practice; application of knowledge to practice, interactive learning	Language barriers – learners/patients and providers; problems of integrating biomedical contents into e-learning curricula, labour-intensive (time, cost) approach	E-learning	Improved learners’ knowledge and self-efficacy, developed confidence in solving clinical/health related problems related to learning in practice, interactive learning between learners and tutors
Khasawneh 2015 [54] USA	E-learning and paediatric education	Descriptive – quantitative study; Wilcoxon rank sum tests	UG 3rd year medical students (*n* = 67)	Use of technology in education	Autonomy, flexibility, reflective thinking, self-confidence and satisfaction	None discussed	E-learning	Improved medical students’ performance, self-confidence in learning, improved satisfaction
Kitching 2015 [55] Australia	Web-based/e-learning and education (medical)	Qualitative; framework analysis	Senior stakeholders from nursing home sector (*N* = 25)	Management policy, learning management systems in HSE.	Change in learning and clinical practice through considering learners’ individuality – background, experiencing support in learning, access to and diversity of information	Poorly considered learners’ needs and interest in learning curricula, poor reflection of current and future workforce needs and demographics, time constraints, inappropriate equipment for technologically illiterate learners	Web-based social media	E-learning is associated with enhanced learning and engagement, thereby positively changing in clinical practice; offered currency and practice contributing to contemporary information; considered learners’ individuality – background, experiencing support in learning; delivered using various styles and formats; access to and diversity of information, meeting learners’ current and future needs
Kokol 2006 [56] Slovenia and USA	E-learning	Mixed methods approach	Full-time and part-time students (*n* = 125)	National and international policy changes in education and learning including advancement of science and technology, including IT	Credibility and motivation, and accessibility. Lifelong learning, promising alternative mode of delivery	Issues related to self-control, inappropriate learning for full-time learners due to their work and personal commitments, lack of knowledge in IT, limited interaction between learners and tutors, and poor access to resources. E-learning is not for all, as some learners are naturally technology-phobic	E-learning	Improved quality of education and integration/application of theoretical context in practice; offered multidisciplinary users.
Morente 2013 [57] Spain	E-learning tool and education on pressure ulcer	RCT - t-test and chi-square tests	Nurses (n = 60; 30 received traditional education; 30- Computer-assisted training)	Influence of technological advancement, promising alternative method to traditional teaching	Flexibility, easy access to materials, immediate feedback from the tutors and/or peers, generate interest, satisfaction	None discussed	E-learning	Reported as an effective and valuable educational tool, positive impact on clinical decision-making process, better learning acquisition, improved education efficiency
Morton 2016 [58] UK	Blended learning	Mixed methods approach	Intercalated BSc 4th year medical students (*n* = 26), F (n = 12)	Increasingly use of e-learning in UG medical education, interactive, more student-centred learning in medical education	Appropriate to teach basic knowledge, interactive component of e-learning	Problems related to learners’ engagement with online self-directed learning	Blended learning	Improved computer literacy, high level of satisfaction, improved medical students’ engagement, improved understanding, efficient approach to learning
Moule 2010 [59] UK	E-learning and students’ experience	Mixed methods approach	Staff (*n* = 35) and students (*n* = 41) from 93 HEIs.	Education policy development and advancement in technological developments in HEIs, constructivist (learner-focused) approach in healthcare education	Flexibility, motivation, and engagement in learning, relevance to practice	Poor access to computers in workplace, limited IT skills as well as poor peer commitments	E-learning	Improved motivation to learning, student-centred learning and engagement.
Naeem 2019 [60] Pakistan	Challenges in blended course	Qualitative design, framework analysis	Postgraduate healthcare students (n = 22)	Institutional support	Flexible, feasible, self-regulation and self-directed learning	Poor instructional design, limited resource provision, poor admin support, poor financial position, poor feedback, issue of time-management,	Blended learning	Enhanced students’ learning; Identified needs of the stakeholder and students
Ota 2018 [61] Australia	Nursing students’ perceptions and challenges to blended study	Quantitative design	UG nursing students (*n* = 109)	Technology in higher education	Autonomy and accountability	Technical difficulties, lack of flexibility, unexpected workload, difficult to link theoretical aspects in practice e.g. real life clinical context	Blended on-line learning	Enhanced students’ motivation and learn
Padalino 2007 [62] Brazil	E-learning and knowledge apprehension	True experimental design- ANOVA tests	Occupational physicians (*n* = 74)	Technology revolution, strategic changes in HE, support CPDs and collaborative learning	Effective and efficient learning, flexibility, cost- and time-saving, provides more individualised learning, adjusted learners’ rhythm	None discussed	E-learning	Obtained higher score, e-learning strategy reported equally effective learning approach, provided opportunities for both tutors and learners by using both technical and pedagogic teaching methods and tools
Parry 2007 [63] UK	E-learning and bioscience	Quantitative; Kruskal-Wallis one-way ANOVA	UG students (*n* = 124; 71 – first year, 33- s year, 20- third year)	Quality of student experience, demands of the programme, changing, i.e. use of VLEs in HE environment	Flexible, convenient and efficient in time – off-campus, access to learning materials (anytime, anywhere). Feedback (formative) received from tutors/peers ‘open dialogue’ found useful and motivating	Lack of engagement due to poor internet access and technical difficulties	E-learning	Increased the depth and breadth of learning, utilised a new method of learning, independent or self-learning, found useful and motivating, developed confidence and promoted engagement
Sinacori 2019 [64] USA	Experiences of nurse educators to online learning	Qualitative design	Nurse educators (*n* = 8)	Faculty development, professional development, contents organisations	Learning new pedagogy, facilitating learning, interaction between students and staff	Lack of professional development, lack of knowledge and online pedagogy, poor learning management system	E-learning	Enhanced students’ motivation and self-regulation.

## Discussion

To the best of our knowledge, this is the first systemic literature review examining and synthesising the factors – enablers or barriers – evidencing to e-learning in making HSE effective. In this study, analysing from 24 unique papers, we found that e-learning has some impact on enhancing learning and performance due to the nature of its flexibility and accessibility. Some evidences indicated that e-learning offers to meet lifelong education needs, as well as widening participation in achieving desired learners’ outcomes in practice, as e-learning approaches are often context-specific.

There are also opportunities for the provision of online access to learning resources and materials development, as well as some opportunities for collaborating and use of open-source materials [59, 68]. Such collaboration or interaction between learners and facilitators would influence an attitude of sharing knowledge, which is one of the crucial elements of e-learning’s *shared enterprise* [69, 70]. Gulati [71] further notes that e-learning often takes place through the reflection of workplace learning; therefore it might be viewed as constructivist e-learning. Holmes and Garder [1] referred to it as *communal constructivism,* arguing that within such a learning context and environment, “each member [learner or facilitator or both] learns with and from others, and contributes learning resources to others”.

The study also found that though e-learning facilitates the process of learning and thereby changes in practice by supporting instructional design and delivery mechanisms, which captures the developing of materials using set learning objectives, including teaching strategies – embedding feedback and evaluation [30] to influence learners’ intrinsic and extrinsic motivation factors, the process has been influenced by several internal, external and contextual factors, including time, IT, flexibility, independence and learners’ motivation and expectations [44, 53, 55].

With reference to medical education, it has been argued that e-learning is primarily meant “to improve the efficiency and effectiveness of educational interventions in the face of the social, scientific, and pedagogic challenges” [22]. Similarly, the study has noted that learning is very much a social phenomenon where interaction and collaboration between learners and tutors embedding feedback and peer support would be an important process that fosters *academic dialogue* between peers and facilitators [72–74]. As Ramsden [75] argues, “learning is about integrating and understanding reality in a different way”. Another interesting point that this study has highlighted is the important role of el-HSE in meeting the demand for “diverse needs of the healthcare employers and individuals by providing education that is flexible, learner-centred, customer-focused”, and with individualised learning and styles, including collaboration, costs and integration [76].

This research further explored the importance of utilising user-friendly IT in online teaching and learning, as this can help achieve changes in competence, performance, and outcomes. This review, however, highlighted a number of underlying challenges. In Gilchrist and Ward’s [77] view, there are several factors, including appropriate policies and strategies, adequate resources and trained staff in place that might interplay in making e-learning or online education effective. One criticism was that e-learning is often viewed as a *technology rather than pedagogy*, as it mostly drives learning through technology, compromising the needs and expectations of learners [78]. Another criticism was that, though the effectiveness of e-learning was documented in other studies conducted, it is still difficult to determine the impact because of the variability in instructional designs [32].

One of the greatest threats to improving e-learning is the impacts of education and performance, particularly in relation to successful course delivery, many of which are challenges in HSE [70, 79].

Similarly, as Schmidt and Gallegos [79] highlight, “[s]uccessful conversion of course delivery method is not always guaranteed” as it has several challenges, including quality of contents, IT and the type/nature of e-learning, which has been discussed earlier. Though e-learning or online education has served well in fostering learning through engaging learners as well as sustaining the growth of the education industry for the last few decades, the review noted that the rapid expansion of the approaches in many parts of the world brings another significant challenge [30, 80, 81]. First, some courses by their nature, e.g. biomedicine and engineering, are more theory-driven, where learners demonstrate their learning and performance in the workplace or in practice. In such a context, e-learning may not be an appropriate approach to learning. Second, mostly in clinical research courses, learners need to develop or learn their level of knowledge and skills through trials, e.g. RCTs, where the constant presence of facilitators or tutors is recommended (or sometimes it might be mandatory). Third, faculty and skills development needs of e-learning among staff or facilitators. Finally, there is a lack of inadequate IT facilities in some educational institutions.

This study argues that while in principle, we can equal or even improve on classical/traditional learning methods in terms of quality of education and increasing learners’ knowledge achievement [13, 53], in practice, learners or facilitators have to face different challenges or debates [82].

Though the study acknowledged the importance of integrating the strengths of synchronous (face-to-face) and asynchronous (text-based internet) learning activities into curricula using two different models or approaches, e.g. (a) blended learning and (b) flipped classroom to facilitate a simultaneous independent and collaborative learning experience due to its flexible and technologically rich format [6, 7], it equally brings a “fundamental reconceptualization and reorganization of the teaching and learning dynamic, starting with various specific contextual needs and contingencies” [7]. Nevertheless, the role of teachers in these models would be crucial, acting as facilitators to support not only students’ subject knowledge, but also their acquisition of skills, qualities and competencies [83].

While analysing e-learning models, designs and components [6–8], it has been seen that for online learning, use of electronic communication and techniques unique to computers is a tool, not unlike the telephone, or postal mail, or even the chalkboard, and which should be evaluated or assessed only in the context of the educational design and relevant values deriving from the local political, cultural, professional and social contexts for situated learning [84]. Any educational tools or models can be used well or used poorly considering the values, beliefs and choices – but that is not the fault of the tools or models. Of course, it also depends on the nature and context of the field as well.

The findings of this research have answered the research objectives, which were to systematically gather and synthesise the evidence around e-learning and the factors – enablers and challenges – associated with making HSE effective. These objectives were achieved by demonstrating the aspects of measures or findings in 24 studies (Table 3). Similarly, the conceptual frameworks developed based on the study and components or elements of el-HSE were identified and discussed, and they were sufficiently described or recorded in the 24 studies to demonstrate a clear link between e-learning and its delivery mechanism and potential learning outcomes or impacts (Fig. 2). In that sense, this review study has clearly provided useful information to policy-planners, educators and decision-makers and other stakeholders in terms of the selection of appropriate methods, mechanisms and tools for education and learning [8].

### Limitations

This systematic review has some limitations. First, efforts were undertaken to identify all relevant articles associated with the enablers and barriers related to e-learning with different disciplines in health science education, using seven well-known electronic databases. No grey literature was searched, thus studies could have been missed. Second, we did not contact any author to ask for additional data/relevant studies that may lead to an important source of publication bias. Third, the identified research studies were variable in quality, sample size and study population. Though the overall methodological quality of the included papers was good, the majority of the included papers (17 of 24) failed to describe appropriate detailed descriptions of sample and sampling procedures [3, 42–45, 47–51, 53, 54, 56–59, 63]. About one-third of the studies (seven of 24) provided inadequate descriptions of methodologies [3, 42, 47, 48, 54, 59, 63].

Given these methodological weaknesses, these were open to bias. Due to the heterogeneity of data, meta-analysis was not possible to measure the effect size of e-learning on health sciences education or the strengths of relationships [85]. Finally, this research study was unfunded, and both time- and resource-limited.

### Strengths

In light of the identified limitations or challenges with robust descriptive literature review data, one of the major strengths of this study was the approach to the literature reviewing/examining from the conceptual framework and its focus on the aspects of making e-learning, in HSE, effective. This study was conducted using a comprehensive search strategy and detailed data extraction method. Similarly, the study has shown that e-learning education can help promote lifelong learning and widening participation in achieving desired learner outcomes – embedding national policy and local context, development of appropriate resources, and collaboration and networking with other providers.

The outcome of this review has revealed several policies and programmatic implications, and the potential benefit could be summarised into two parts: first, this will help learners and facilitators or instructors as well as other stakeholders to better understand the issues related to barriers and/or facilitators associated with e-learning, and second, this study will help healthcare education policy-planners and decision-makers understand the conditions or factors that may facilitate or constrain e-learning, so that they would be able to implement e-learning policy more effectively in the HSE context. In addition, this study would contribute to developing appropriate policies, guidance and initiatives within the context of theoretical perspectives (education and culture) to confirm or extend the significance of e-learning to establish when, how and where it fits best for making HSE effective.

Though online learning has continued to grow in undergraduate, postgraduate and CPD, there is no doubt that this would need to structure the learning experience to ensure that effective online discourse occurs between the various members of this online discussion (including facilitators, experts, and practitioners) so that the complexity of practice can be grasped and practical solutions proposed, implemented and refined in terms of meeting the emerging competencies [11]]. At the same time, many face-to-face classroom teachers need to learn new knowledge/skills to adapt the current teaching to e-learning education [see 25].

## Conclusion

This review explores the potential role of e-learning in general and in HSE in particular, examining the factors – enablers or challenges – using systemic literature review, which has revealed this as a less-studied area of research. The available evidence suggests that making e-learning effective in the health sciences is affected not only by the lack of resources – significant time and cost savings and support, but also that design aspects should be taken into account in creating or promoting self-directed learning. At the same time, appropriate development of institution strategies is paramount. This could include such elements as flexibility and access, learning styles, costs, and integration to promote learners’ knowledge and understanding evidence-based national drivers and local contexts, putting learners’ learning experience as the main driver, rather than IT, in practice.

The outcome of this review has suggested that el-HSE, both academic and professional, or CPD training and education in the workplace, have the potential to improve learners’ level of knowledge and performance through making HSE learning resources accessible to learners or facilitators, regardless of their geographical locations and timescale. This study, therefore, suggests that to bring a positive change in learning and practice, we need to put learners at the centre of learning – considering the pedagogic design, learning styles and their expectations, integrating e-learning into health science education curriculum and practice. Further studies are needed to ensure rigorous study design to deliver quality and effective e-learning, use of technology advancement in healthcare research education for all practising healthcare professionals in education-related randomised controlled trials, and blinding in HSE.

## Supplementary information

**Additional file 1.** Systematic review protocol.

**Additional file 2.** Critical appraisal of included studies (QualSyst tools).

**Additional file 3.** Excluded studies.

## Data Availability

All raw data used in this systematic review were extracted from published articles.

## Abbreviations

HSE: Health Sciences Education
SLR: Systemic Literature Review
IT: Information Technology
AMED: Allied & Complementary Medicine
DH-DATA: Department of Health Data
CINAHL: Cumulative Index to Nursing and Allied Health Literature
TA: Thematic Analysis
CPD: Continuing Professional Development
UG: Undergraduate
PG: Postgraduate
RCT: Randomised Controlled Trial
CF: Conceptual Framework
