# Supplementary material for: A systematic review of the factors – enablers and barriers – affecting e-learning in health sciences education
Source: BMC Med Educ. 2020 Mar 30;20:91. doi: 10.1186/s12909-020-02007-6 (PMC7106784; doi:10.1186/s12909-020-02007-6)
Supplement: Supplementary file 1 — Additional file 1. Systematic review protocol. [file 12909_2020_2007_MOESM1_ESM.docx]

**Additional file 1: Systematic review protocol**

**A systematic review of the factors – enablers and barriers – affecting e-learning in health sciences education**

**1. Background**

E-learning has been defined as “an educational method that facilitates learning by the application of information technology and communication providing an opportunity for learners to have access to all the required education programmes” [1]. Recently, much attention has been given to e-learning in higher education as it provides better access to learning resources online, utilising technology – regardless of learners’ geographical locations and timescale – to enhance learning. Though some published systemic reviews on e-learning have provided some promises that e-learning would be equally as effective as traditional methods of learning or teaching, still there is very limited evidence demonstrating when and how best e-learning enhances education and learning, and the factors associated with it [2-6].

## **2. Objectives and research question**

The objective of this review is to conduct a systematic review to identify and synthesise the factors – enablers and barriers – affecting e-learning in health sciences education (el-HSE) that have been reported in the medical literature.

***Review question***

This systematic review will be guided by this following research question: What are the factors – enablers and barriers – affecting e-learning in health sciences education (el-HSE) as reported in studies from 1980 to 2019?

## **3. Methods**

We will conduct a systematic review research of both randomised controlled trials and non-randomised controlled trials. Seven databases – MEDLINE, EMBASE, Allied & Complementary Medicine, DH-DATA, PsycINFO, CINAHL, and Global Health – will be searched. Primary search terms are e-learning (all synonyms) and health sciences education (all synonyms) using ‘Textword searching’, and ‘Thesaurus (MeSH, EMTREE) terms. The systematic review methodology will adhere to the *Preferred* Reporting *Items for Systematic Reviews and Meta-Analyses* (PRISMA) statement [7].

### *3.1 Key terms:*

The following key terms and concepts relate to this systematic literature review, their definitions having been adapted from these sources:

- E-learning: e-learning is defined as [T]he use of the internet to access learning materials; to interact with the content, instructor, and other learners; and to obtain support during the learning process, in order to acquire knowledge, to construct personal meaning, and to grow from the learning experience [8].
- European e-learning Action Plan has also defined e-learning focusing on similar attributes, defining it as the use of new multimedia technologies and the Internet to improve the quality of learning by facilitating access to resources and services as well as remote exchanges and collaboration [9].
- Distributed learning - Distributed learning is an approach that allows students and faculty to enter the learning environment at different times and from different locations [10].

### *3.2 Search strategy and search terms*

This literature review will use peer-reviewed literature, which will be located using the following seven electronic bibliographic databases: MEDLINE, EMBASE, Allied & Complementary Medicine, DH-DATA, PsycINFO, CINAHL, and Global Health.

The search structure will consist of the following:

- terms related to e-learning;
- AND terms related to continuing medical education or health sciences education;
- AND terms related to challenges;
- AND terms related to enablers including drivers or facilitators.

The search terms used for the main bibliographic databases are given in Section 4.

Searches will also be supplemented by reviewing the reference lists (‘references of references’) of selected articles to find any other relevant papers.

### *3.3 Inclusion/exclusion criteria*

The following seven key inclusion criteria will be used in this review:

- Types of studies: Primary qualitative and quantitative research studies. Study designs including randomised controlled trials, non-randomised controlled trials, controlled before-after studies, controlled interrupted time series studies.
- Focus of studies: E-learning implemented in health sciences education, investigation of the enablers and barriers about el-HSE related to learning performance or outcomes.
- Data type(s): Must include primary data.
- Date of intervention and publication: 1980 to 2019.
- Publication language: English.

The following criteria will be used to exclude studies from this review:

- Studies with no specific e-learning in health sciences education and no outcomes (i.e., excluding studies that do not examine enablers and barriers).
- Review papers; only references listed in review papers will be screened to find more primary data sources.

### *3.4 Study screening and data extraction*

The systematic literature review will be conducted by two reviewers (KR and LJ) for quality assurance. Discrepancies in reviewer selections will be resolved at a meeting between reviewers prior to selected articles being retrieved.

Data will be screened with the following five stages:

1. Electronic database search using terms; with results imported into reference management software, and duplicates removed.
2. Title and abstract reviewed to remove studies not meeting the inclusion criteria.
3. Manuscript review to remove studies that do not meet inclusion criteria; paper selection.
4. Review of references of selected papers.
5. Final paper selection, data extraction, and quality assessment.

Data will be extracted based on the specific points noted below and input into a standardised Excel database (adopted from Soklaridis [11]).

- Citation - study authors or agency, year
- Study country
- Aim and purpose
- Discipline of researcher(s): medical, nursing, physicians, undergraduate, post-graduate medical/nursing/allied healthcare students, primary care
- Population type: HEIs students/leaners, staff
- Research design
- Theoretical framework/approach
- Measurement outcomes (e.g. enablers and barriers including potential including factors, delivery mechanisms, wider impacts)
- Data collection
- Data analysis
- Research strengths from the literature
- Research gaps from the literature

### *3.5 Data categorisation and analysis*

Data findings will be organised in relation to the key issues of quantity and quality of the evidence base. To increase clarity of the final results, the studies selected will be arranged using Table 1 and Table 2.

**Table 1: Quality review criteria** (adapted from Lee [12] and Maharaj [13])

| **Criteria for quantitative studies** | **Criteria for qualitative studies** |
| --- | --- |
| 1. Question / objective sufficiently described?  2. Study design evident and appropriate?  3. Method of subject/comparison group selection or source of information/input variables described and appropriate?  4. Subject (and comparison group, if applicable) characteristics sufficiently described?  5. If interventional and random allocation was possible, was it described?  6. If interventional and blinding of investigators was possible, was it reported?  7. If interventional and blinding of subjects was possible, was it reported?  8. Outcome and (if applicable) exposure measure(s) well defined and robust to measurement / misclassification bias? means of assessment reported?  9. Sample size appropriate?  10. Analytic methods described/justified and appropriate?  11. Some estimate of variance is reported for the main results?  12. Controlled for confounding?  13. Results reported in sufficient detail?  14. Conclusions supported by the results? | 1. Question / objective sufficiently described?  2. Study design evident and appropriate?  3. Context for the study clear?  4. Connection to a theoretical framework / wider body of knowledge?  5. Sampling strategy described, relevant and justified?  6. Data collection methods clearly described and systematic?  7. Data analysis clearly described and systematic?  8. Use of verification procedure(s) to establish credibility?  9. Conclusions supported by the results?  10. Reflexivity of the account? |

**Table 2: Quality assessment corresponding to adapted Lee [12] and Maharaj [13] criteria**

| **Level of Quality** | **Rating of Evidence** |
| --- | --- |
| **Quantitative studies** | |
| STRONG | Summary score of >0.80 |
| GOOD | Summary score of 0.71-0.79 |
| ADEQUATE | Summary score of 0.50-0.70 |
| LIMITED | Summary score of <0.50 |
| **Qualitative studies** | |
| ADEQUATE | Summary score of ≥0.55 |
| Low quality | Summary score of ≤0.54 |

(A cut-off of 75% as the threshold for quantitative and 55% for qualitative papers will be set up)

## **4. Search terms used for key bibliographic databases**

***Sources:***

Published literature: MEDLINE, EMBASE, Allied & Complementary Medicine, DH-DATA, PsycINFO, CINAHL, and Global Health.

**MEDLINE Search strategy**

(Similar terms under each concept will be modified as needed for use in other databases)

***Concept 1 - E-learning:***

"e-learning"[All Fields] OR "education, distance"[MeSH Terms] OR "online learning"[All Fields] OR "online instruction"[All Fields] OR "distance education"[All Fields] OR "distance learning"[All Fields] OR "blended learning"[All Fields] OR "pure learning"[All Fields] OR "computer-assisted instruction"[MeSH Terms] OR "computer assisted instruction"[All Fields] OR "electronic-learning"[All Fields] OR "computer based training"[All Fields] OR "libraries, digital"[MeSH Terms] OR "digital libraries"[All Fields] OR "mobile learning"[All Fields] OR "electronic education"[All Fields] OR "online instruction"[All Fields] OR "distance teaching"[All Fields] OR "distributed learning"[All Fields] OR "virtual patients"[All Fields] OR "virtual microscopy"[All Fields] OR "virtual environment"[All Fields] OR "virtual learning"[All Fields] OR "Objective Structured Clinical Exams"[All Fields] OR "web-based learning"[All Fields] OR "internet-based learning" [All Fields] OR "multi-media learning"[All Fields] OR "technology-enhanced learning"[All Fields]

***Concept 2 - Health Sciences Education:***

"continuing medical education"[All Fields] OR "education, medical, continuing"[MeSH Terms] OR "Continuing professional development"[All Fields] OR "medical education"[All Fields] OR "education, medical"[MeSH Terms] OR "health sciences"[All Fields] OR "Basic Sciences"[All Fields] OR “health” OR "health"[MeSH Terms] OR "public health education"[All Fields] OR "public health"[All Fields] OR "public health"[MeSH Terms] OR "nursing education"[All Fields] OR "education, nursing"[MeSH Terms] OR "public health nursing"[All Fields] OR "public health nursing"[MeSH Terms] OR "allied health education"[All Fields] OR "health education"[All Fields] OR "health education"[MeSH Terms] OR "primary health care"[All Fields] OR "primary health care"[MeSH Terms] OR "evidence-based medicine"[All Fields] OR "evidence-based medicine"[MeSH] OR "general practice"[MeSH Terms] OR "general practice"[All Fields]

***Concept 3 - Enablers:***

"Enablers" [All Fields] OR "drivers" [All Fields] OR "facilitators" [All Fields]

***Concept 4 - Barriers*:**

"Challenges"[All Fields]) OR "barriers" [All Fields] OR "obstacles" [All Fields] OR "constraints" [All Fields]) OR "hinders" [All Fields]

We will combine these four concepts so that all concepts are in the same references.

**References**

1. Golband F, Hosseini AF, Mojtahedzadeh R, Mirhosseini F, Bigdeli S. The correlation between effective factors of e-learning and demographic variables in a post-graduate program of virtual medical education in Tehran University of medical sciences. Acta Med Iran. 2014; 52:860-64.
2. Cook D, Levinson A, Garside S, Dupras D, Erwin P, Montori V. Internet-based learning in the health professions: a meta-analysis. JAMA. 2008; 300:1181-96.
3. Childs S, Blenkinsopp E, Hall A, Walton G. Effective e-learning for health professionals and students – barriers and their solutions. A systematic review of the literature – findings from the HeXL project. Health Info Libr J. 2005; 22:20-32.
4. Curran VR, Fleet L. A review of evaluation outcomes of web-based continuing medical education. Med Educ. 2005; 39:561-67.
5. McCutcheon K, Lohan M, Traynor M, Martin D. A systematic review evaluating the impact of online or blended learning vs. face-to-face learning of clinical skills in undergraduate nurse education. J Adv Nurs. 2014; 71:255-70.
6. Wutoh R, Boren SA, Balas EA. E-learning: a review of internet-based continuing medical education. J Contin Educ Health Prof. 2004; 24:20-30.
7. Moher D, Liberati A, Tetzlaff J, Altman DG. The PRISMA Group. Preferred reporting items for systematic reviews and meta-analyses: the PRISMA statement. PLoS Med. 2009: 6: e1000097. doi:10.1371/journal.pmed1000097.
8. Anderson T. Towards a theory of online learning: the theory and practice of online learning. Edmonton: AU Press, Athabasca University; 2008.
9. Commission of the European Communities. The e-learning action plan: designing tomorrow’s education. 2001. http://www.aic.lv/bolona/Bologna/contrib/EU/e-learn_ACPL.pdf. Accessed 30 Oct 2018.
10. Oblinger DG, Maruyama MK. Distributed learning. Cause professional paper series, # 14. Colorado: Cause; 1996.
11. Soklaridis S, Ferguson G, Bonato S, et al. Being there: protocol for a scoping review of the medical education literature on grief support training for medical professionals. BMJ Open 2018; 8:e022778. doi:10.1136/bmjopen-2018-022778
12. Lee L, Packer TL, Tang SH, Girdler S. Self-management education programs for age-related macular degeneration: a systematic review. Australas J Ageing 2008: 27:170-76.
13. Maharaj S, Harding R. The needs, models of care, interventions and outcomes of palliative care in the Caribbean: a systematic review of the Evidence. BMC Palliat Care 2016:15:9. doi:10.1186/s12904-016-0079-6.
