# Supplementary material for: A systematic review of the factors – enablers and barriers – affecting e-learning in health sciences education
Source: BMC Med Educ. 2020 Mar 30;20:91. doi: 10.1186/s12909-020-02007-6 (PMC7106784; doi:10.1186/s12909-020-02007-6)
Supplement: Supplementary file 2 — Additional file 2. Critical appraisal of included studies (QualSyst tools). [file 12909_2020_2007_MOESM2_ESM.doc]

Additional file 2: Critical appraisal of included studies

[2-YES, 1-PARTIAL -1, 0 - NO, NA- NOT APPLICABLE]

| Quantitative studies | | | | | | | | | | | | | | | | | | | | | Overall Quality score | |
| --- | --- | --- | --- | --- | --- | --- | --- | --- | --- | --- | --- | --- | --- | --- | --- | --- | --- | --- | --- | --- | --- | --- |
| Checklist/ First author and year | 1 Question / objective sufficiently described? | 2 Study design evident and appropriate? | 3 Method of subject/comparison group selection or source of  information/input variables described and appropriate? | 4 Subject (and comparison group, if applicable) characteristics  sufficiently described? | 5 If interventional and random allocation was possible, was it  described? | 6 If interventional and blinding of investigators was possible, was it  reported? | 7 If interventional and blinding of subjects was possible, was it  reported? | 8 Outcome and (if applicable) exposure measure(s) well defined  and robust to measurement / misclassification bias? means of  assessment reported? | 9 Sample size appropriate? | 10 Analytic methods described/justified and appropriate? | 11 Some estimate of variance is reported for the main results? | | 12 Controlled for confounding? | | 13 Results reported in sufficient detail? | | 14 Conclusions supported by the results? | | Summary score | |
| Golband 2014 [3] | 2 | 2 | 1 | 1 | NA | NA | NA | 2 | 1  not describe how they selected samples | 2 | 2 | | NA | | 1 | | 2 | | Total sum (16)  Total possible sum (20) | | Quant 0.8 | |
| Gensichen 2009 [50] | 2 | 1 | 1 | 1 | NA | NA | NA | 2 | 1 not describe how they selected samples | 1 | 2 | | NA | | 2 | | 2 | | Total sum (15)  Total possible sum (20) | | Quant 0.75 | |
| Ikram 2015 [56] | 2 | 2 | 2 | 2 | NA | NA | NA | 2 | 1 | 2 | 2 | | NA | | 2 | | 2 | | Total sum (19)  Total possible sum (20) | | Quant 0.95 | |
| Hawthrone 2009 [54] | 1 | 2 | 2 | 2 | NA | NA | NA | 2 | 2 | 2 | 2 | | NA | | 2 | | 2 | | Total sum (19)  Total possible sum (20) | | Quant 0.95 | |
| Khasawneh 2015 [57] | 2 | 2 | 1 | 2 | NA | NA | NA | 1 | 1 not describe how they selected samples | 2 | 1 | | NA | | 1 | | 2 | | Total sum (15)  Total possible sum (20) | | Quant 0.75 | |
| Hadadgar 2016 [52] | 2 | 2 | 2 | 2 | NA | NA | NA | 2 | 1 not describe how they selected samples | 2 | 2 | | NA | | 2 | | 2 | | Total sum (19)  Total possible sum (20) | | Quant 0.95 | |
| Beckman 2008 [45] | 2 | 2 | 1 (no s. frame) | 2 | NA | NA | NA | 2 | 1 (provided v basic info) | 2 | 2 | | NA | | 2 | | 2 | | Total sum (18)  Total possible sum (20) | | Quant 0.90 | |
| Parry 2007 [66] | 1 | 1 | 1 | 2 | NA | NA | NA | 2 | 1(provided v basic info) | 1 | 2 | | NA | | 2 | | 2 | | Total sum (15)  Total possible sum (20) | | Quant 0.75 | |
| Gormley 2009 [51] | 2 | 1 | 1 | 2 | NA | NA | NA | 2 | 1(provided v basic info) | 2 | 2 | | NA | | 2 | | 2 | | Total sum (17)  Total possible sum (20) | | Quant 0.85 | |
| Boye 2012 [46] | 2 | 2 | 1 | 2 | NA | NA | NA | 2 | 1 | 1 | 2 | | NA | | 2 | | 2 | | Total sum (17)  Total possible sum (20) | | Quant 0.85 | |
| Morente 2013 [60] | 2 | 2 | 2 | 2 | 1 | 1 | 1 | 2 | 1 | 2 | 2 | | 1 | | 2 | | 2 | | Total sum (23)  Total possible sum (28) | | Quant 0.82 | |
| Padalino 2007 [65] | 2 | 2 | 2 | 1 | 2 | 1 | 1 | 2 | 2 | 2 | 2 | | 2 | | 2 | | 2 | | Total sum (25)  Total possible sum (28) | | Quant 0.89 | |
| Hugenholtz 2008 [55] | 2 | 1 | 2 | 2 | 2 | 1 | 1 | 1 | 1 | 2 | 2 | | 1 | | 2 | | 1 | | Total sum (21)  Total possible sum (28) | | Quant 0.75 | |
| Ota 2018 [64] | 2 | 2 | 2 | 1 | NA | NA | NA | 1 outcomes not provided in details | 2 | 1 nothing justified why authors used stat to analysis these data | 1 | | NA | | 2 | | 1 | | Total sum (15)  Total possible sum (20) | | Quant 0.75 | |
| Qualitative studies | | | | | | | | | | | | | | | | | | | | | | |
| Checklist: | 1 Question / objective sufficiently described? | 2 Study design evident and appropriate? | 3 Context for the study clear? | 4 Connection to a theoretical framework / wider body of knowledge? | 5 Sampling strategy described, relevant and justified? | 6 Data collection methods clearly described and systematic? | 7 Data analysis clearly described and systematic? | 8 Use of verification procedure(s) to establish credibility? | 9 Conclusions supported by the results? | 10 Reflexivity of the account? | |  | |  | |  | |  | | Total sum  Total possible sum  Summary score | | Quality score |
| Hammarlund2015 [53] | 1 | 1 | 2 | 2 | 1 | 1 | 2 | 2 | 2 | 2 | |  | |  | |  | |  | | Total sum (16)  Total possible sum (20) | | Quali 0.80 |
| Kitching 2015 [58] | 2 | 2 | 2 | 1 | 2 | 2 | 2 | 2 | 2 | 2 | |  | |  | |  | |  | | Total sum (19)  Total possible sum (20) | | Quali 0.95 |
| Gagnon 2007 [48] | 2 | 1 | 2 | 2 | 1 | 2 | 2 | 1 | 2 | 1 | |  | |  | |  | |  | | Total sum (16)  Total possible sum (20) | | Quali 0.80 |
| Docherty 2006 [47] | 2 | 2 | 2 | 1 | 1 | 2 | 2 | 1 | 2 | 2 | |  | |  | |  | |  | | Total sum (17)  Total possible sum (20) | | Quali 0.85 |
| Gardner 2016 [49] | 2 | 1 | 2 | 2 | 2 | 2 | 2 | 2 | 2 | 2 | |  | |  | |  | |  | | Total sum (19)  Total possible sum (20) | | Quali 0.95 |
| Naeem 2019 [63] | 2 | 2 | 2 | 2 | 1 | 2 | 1 | 2 | 1 | 0 | |  | |  | |  | |  | | Total sum (15)  Total possible sum (20) | | Quali 0.75 |
| Sinacori 2019 [67] | 2 | 2 | 2 | 1 | 1 | 2 | 1 | 1 | 2 | 1 | |  | |  | |  | |  | | Total sum (15)  Total possible sum (20) | | Quali 0.75 |
| Mixed methods | | | | | | | | | | | | | | | | | | | | | | |
| Quantitative | 1 Question / objective sufficiently described? | 2 Study design evident and appropriate? | 3 Method of subject/comparison group selection or source of  information/input variables described and appropriate? | 4 Subject (and comparison group, if applicable) characteristics  sufficiently described? | 5 If interventional and random allocation was possible, was it  described? | 6 If interventional and blinding of investigators was possible, was it  reported? | 7 If interventional and blinding of subjects was possible, was it  reported? | 8 Outcome and (if applicable) exposure measure(s) well defined  and robust to measurement / misclassification bias? means of  assessment reported? | 9 Sample size appropriate? | 10 Analytic methods described/justified and appropriate? | | 11 Some estimate of variance is reported for the main results? | | 12 Controlled for confounding? | | 13 Results reported in sufficient detail? | | 14 Conclusions supported by the results? | |  | | Quality score |
| Qualitative | 1 Question / objective sufficiently described? | 2 Study design evident and appropriate? | 3 Context for the study clear? | 4 Connection to a theoretical framework / wider body of knowledge? | 5 Sampling strategy described, relevant and justified? | 6 Data collection methods clearly described and systematic? | 7 Data analysis clearly described and systematic? | 8 Use of verification procedure(s) to establish credibility? | 9 Conclusions supported by the results? | 10 Reflexivity of the account? | |  | |  | |  | |  | |  | | Quality score |
| Kokol 2006 [59] | 1 | 2 | 2/2 | 2/2 | NA/1 | NA/1 | NA/1 | 1/1 | 1/1 | 1 | | 1 | | na | | 1 | | 1 | | Quanti. 13/20  Quali: 13/20 | | Quant. 0.65  Quali. 0.65 |
| Moule 2010 [62] | 2 | 2 | 2 | 2/2 | NA/1 | NA/2 | NA/2 | 1/2 | 2 | 2 | | 2 | | na | | 2 | | 2 | | Quanti. 19/20  Quali: 17/20 | | Quant. 0.95  Quali. 0.85 |
| Morton 2016 [61] | 2 | 2 | 2 | 1/2 | NA/1 | NA/2 | NA/2 | 2/2 | 1 | 2 | | 2 | | na | | 2 | | 2 | | Quanti. 18/20  Quali: 18/20 | | Quant. 0.90  Quali. 0.90 |
| Quality interpretation | - For quantitative papers: strong (summary score of >0.80), good (summary score of 0.71-0.79),adequate (summary score of 0.50-0.70) and limited (summary score of <0.50), and - For qualitative papers: a score of ≥0.55 as an ‘adequate quality’ paper. A score of ≤0.54 was deemed as a ‘low quality’ paper. | | | | | | | | | | | | | | | | | | | | | |

Source: Lee [43], Maharaj [44]
