# Supplementary material for: A systematic review of the factors – enablers and barriers – affecting e-learning in health sciences education
Source: BMC Med Educ. 2020 Mar 30;20:91. doi: 10.1186/s12909-020-02007-6 (PMC7106784; doi:10.1186/s12909-020-02007-6)
Supplement: Supplementary file 3 — Additional file 3. Excluded studies. [file 12909_2020_2007_MOESM3_ESM.docx]

Additional file 3: Excluded studies

| **Reason for exclusion** | **Number (%)** | **Studies (First author and year)** |
| --- | --- | --- |
| Secondary - review studies | 19 (57.57%) | Arkorful [1]; Cook [2]; Cook [3]; Cook [4]; Cook [5]; Cook [6]; Doherty [7]; Dubois [8]; Frehywot [9]; Jang [10]; Klein [11]; Lateef [12]; Lau [13]; De Leeuw [14]; Vaona [15]; Purkis [16]; Ruiz [17]; Ruiz [18]; Wutoh [19] |
| Studies not related to health/ barriers/enablers | 6 (18.18%) | Allan [20]; Issa [21]; Mirabella [22]; Coryell [23]; Shraim [24]; Njenga [25] |
| Poor methodology | 3 (9.09%) | Luke [26]; Wahl [27]; Walsh [28] |
| Reports | 2 (6.06%) | Golden [29]; Finlayson [30] |
| AMEE Guide Supplements | 2 (6.09%) | Bilham [31]; Rajapakse [32] |
| Commentary | 1 (3.03%) | Smothers [33] |

**References**

1. Arkorful V, Abaidoo N. The role of e-learning, the advantages and disadvantages of its adoption in higher education. International Journal of Education and Research. 2014; 2: 397-410.
2. Cook DA. A practical guide to developing effective web-based learning. J Gen Intern Med. 2004; 19: 698-707.
3. Cook DA. Where are we with Web-based learning in medical education? Med Teach. 2006; 28: 594-98.
4. Cook DA. The research we still are not doing: an agenda for the study of computer-based learning. Acad Med. 2005; 80: 541-48.
5. Cook DA, Levinson AJ, Garside S, Dupras DM, Erwin PJ, Montori VM. Internet- based learning in the health professions: A meta-analysis. JAMA. 2008; 300: 1181-96.
6. Cook DA, McDonald FS. E-learning: Is there anything special about the “e”? Perspect Bio Med. 2008; 51: 5-21.
7. Doherty I, McKimm J. E-learning in clinical teaching. Br J Hosp Med (Lond). 2010; 71: 44-7.
8. Dubois EA, Franson KL. Key steps for integrating a basic science throughout a medical school curriculum using an e-learning approach. Med Teach. 2009; 31: 822-8.
9. Frehywot S,  Vovides Y, Talib Z, Mikhail N, Ross H, Wohltjen H, et al. E-learning in medical education in resource constrained low- and middle-income countries. Hum Resour Health.2013; 11: 4. [doi.org/10.1186/1478-4491-11-4](https://doi.org/10.1186/1478-4491-11-4).
10. Jang KS, Hwang SY, Park SJ, Kim YM, Kim MJ. Effects of a web-based teaching method on undergraduate nursing students’ learning of echocardiography. J Nurs Educ. 2005; 44:35-9.
11. Klein D, Ware M. E-learning: new opportunities in continuing professional development. Learned Publishing. 2003; 16: 34-46.
12. Lateef F. Blended learning in emergency medicine: Implementing the e-learning component. South Asian J Med Educ. 2014; 8: 60-65.
13. Lau F, Bates J. A review of e-learning practices for undergraduate medical education. J Med Syst. 2004; 28: 71-87.
14. De Leeuw RA, Westerman M, Nelson E, Ket JC, Scheele F. Quality specifications in postgraduate medical e-learning: An integrative literature review leading to a postgraduate medical e-learning. BMC Med Educ. 2016; 16: 168.
15. Vaona A, Banzi R, Kwag KH, Rigon G, Cereda D, Pecoraro V, Tramacere I, Moja L. E-learning for health professionals. Cochrane Database Syst Rev. 2018; 1:CD011736. doi: 10.1002/14651858.CD011736.pub2.
16. Purkis N, Gabb CA. Online learning for professional development. Nurs Times. 2013; 109 (51), 16-18.
17. Ruiz JG, Candler C, Qadri S, Roos B. E-learning as evidence of educational scholarship: A survey of chairs of promotion and tenure committees at US medical schools. Acad Med. 2009; 84: 47-57.
18. Ruiz JG, Mintzer MJ, Leipzig RM. The impact of E-learning in medical education. Acad Med.2006; 8: 207-12.
19. Wutoh R, Boren SA, Balas EA. E-learning: a review of internet-based continuing medical education. J Contin Educ Health Prof. 2004; 24: 20-30.
20. Allan S, Jones K, Walker, S. Retrofitting e-learning to an existing distance learning course: a case study. Architectural Engineering and Design Management. 2006; 2: 137-47.
21. Issa N, Schuller M, Santacaterina S, Shapiro M, Wang E, Mayer R, DaRosa DA. Applying multimedia design principles enhances learning in medical education. Med Educ. 2011; 45: 818-26.
22. Mirabella V, Kimani S, Gabrielli S, Catarci T. Accessible e-learning material: a no-frills avenue for didactical experts. New Review of Hypermedia and Multimedia. 2004; 10: 165-80.
23. Coryell JE, Chlup D. Implementing e-learning components with adult English language learners: vital factors and lessons learned. Computer Assisted Language Learning. 2007; 20: 263-78.
24. Shraim K, Khlaif Z. An e-learning approach to secondary education in Palestine: opportunities and challenges. Information Technology for Development. 2010; 16: 159-73.
25. Njenga JK, Fourie LC. The myths about e-learning in higher education. British Journal of Education Technology.2008; 41: 199-212.
26. Luke R, Solomon P, Baptise S, Hall P, Orchard C, Rukholm E, Cater L. Online interprofessional health sciences education: From theory to practice. J Contin Educ Health Prof. 2009; 29: 161-67.
27. Wahl SE, Latayan M. Nursing education innovation: Using e-learning technology to meet learners’ needs. J Contin Educ Nurs. 2011; 41: 483-84.
28. Walsh K. E-learning and simulation in medical education: an opportunity to integrate? Br J Hosp Med (Lond). 2016; 77: 592-93.
29. Golden S, McCrone T, Walker M, Rudd P. Impact of e-learning in further education: Survey of scale and breadth. National Foundation for Education Research. Research Report No. 745. London: DfES; 2006.
30. Finlayson H, Maxwell B, Caillau I, Tomalin J. E-learning in further education: the impact on student intermediate and end-point outcomes. Research Report No. 739. Sheffield: Sheffield Hallam University; 2006.
31. Bilham T. e-Learning in medical education: Guide supplement 32.5- viewpoint. Med Teach. 2009; 31: 449-51.
32. Rajapakse S, Fernando D, Rubasinghe N, Gurusinghe S. E-Learning in medical education: guide supplement 32.6 - practical application. Med Teach. 2009; 31: 452-53.
33. Smothers V, Ellaway R, Greene P. The e-learning evolution – leveraging new technology approaches to advanced healthcare education. Med Teach. 2008; 30: 117-18.
